# Precarious Essential Work, Immigrant Dairy Farmworkers, and Occupational Health Experiences in Vermont

**DOI:** 10.3390/ijerph18073675

**Published:** 2021-04-01

**Authors:** Bindu Panikkar, Mary-Kate Barrett

**Affiliations:** 1 Bindu Panikkar, Environmental Studies Program and the Rubenstein School of the Environment and Natural Resources, University of Vermont, 81 Carrigan Dr., Burlington, VT 05405, USA; 2 College of Agriculture and Life Sciences, University of Vermont, 146 University Place, Morril Hall, Burlington, VT 05405, USA; marykate.barrett1@gmail.com

**Keywords:** dairy workers, immigrant farmworkers, occupational health, precarious work, essential worker

## Abstract

Migrant dairy workers in Vermont face a wide range of occupational and health hazards at work. This research examines the environmental risks, occupational health hazards, and health outcomes experienced by migrant dairy farm workers in Vermont. This research draws on a triangulation of sources including analysis of data—surveys and interviews with migrant dairy farmworkers gathered by the organization Migrant Justice since 2015 as well as relevant key informant interviews with community organizations across the state to characterize the occupational health experiences of migrant dairy workers in Vermont. Our results show that Vermont migrant dairy farmworkers received poor health and safety training and lacked sufficient protective gear. Over three quarters of the respondents reported experiencing harm from chemical and biological risks. Close to half the survey respondents reported headaches, itchy eyes and cough; a quarter reported breathing difficulties; three fourths reported being hurt by animal-related risks. These exposures and existing health concerns are avoidable. Migrant workers require better social representation and advocates to negotiate better work-related protection and training, access to health services, and social welfare to ensure their health and safety.

## 1. Introduction

Dairy industry has one of the highest injuries and fatalities within agricultural industry [1,2,3,4,5,6]. According to the 2019 Census of Fatal Occupational Injuries, dairy cattle and milk production accounted for 33% of the fatalities within animal production and aquaculture [2]. Most dairy industry deaths resulted from machinery, equipment and animal handling in Wisconsin and New York, states with the largest proportion of US dairy worker fatalities [3]. While there has been a decrease in fatalities in the dairy industry, the non-fatal injuries and health outcomes have continued to remain high and unaddressed within the industry [3].

Dairy farm workers are exposed to a wide range of occupational hazards. Livestock farming may release numerous toxic contaminants including bioaerosols, ammonia, hydrogen sulfide gas, particulates, complex mixtures of chemicals, and volatile organic compounds [7,8,9,10]. Bioaerosol emissions in dairy farms can result from animal waste, feed, and bedding material and farmers are exposed to it while handling animals, milking (especially automatic milking), feeding and cleaning. Exposures may vary depending upon the type of flooring, ventilation, heating, and maintenance practices used in the farm [7]. Bioaerosols exposures have shown to cause respiratory disorders, inflammatory reactions, fatigue, weakness, headaches, and gastrointestinal problems in farm workers [7,8,9,10,11]. High levels of bioaerosols are also linked to hypersensitivity pneumonitis, a potentially fatal disease also known as Farmers Lung [7,11]. Chemical exposures among dairy farmworkers may range from volatile organic compounds, pesticides, formaldehyde, heavy metals, and cleaning products [12]. Acute effects from exposure to these chemicals include skin rashes, eye and respiratory irritation, and death. Chronic exposures to these substances have implicated cancer and multi-generational cognitive deficits [11,12,13,14,15,16,17,18,19,20,21]. Exposures to volatile organic compounds, hydrogen sulfide, ammonia, and nitrogen oxides in dairy farms have shown to worsen respiratory illnesses [13,14,15,16,17,18,19]. Formaldehyde, used in footbaths to prevent disease, is a known carcinogen and irritant [21]. Negative health outcomes from air emissions have been noted also among residents living near livestock farms [22,23]. Livestock also pose risk of zoonotic infectious diseases among farm workers [24,25] and exposure to antibiotic-resistant bacteria due to the increased use of antibiotics and antimicrobials in dairy cattle [26,27]. Safety interventions have been shown to be successful in minimizing health risks [3,28,29,30,31,32], however, many scholars emphasize that few farms have safety expertise, worker safety training, and health insurance, and dairy farming injuries remain common [1,3,4].

Immigrant workers make up an estimated 51% of all labor in the United States dairy industry and over 79% of the milk produced in the US come from farms that employ immigrant workers [3,33,34]. These workers are predominantly Spanish speakers [3,33]. These immigrant workers are integral to the economic sustainability of the dairy industry in the US, yet they are the most vulnerable to environmental and occupational exposures and poor health outcomes, occupational injuries and fatalities in the dairy industry compared to white workers [3,4,30,35]. Still, few studies have characterized the work experiences, day to day exposures, and health outcomes of these workers in the dairy industry [35]. Here, we examine the occupational risks and health experiences of migrant dairy farmworkers in Vermont.

Vermont is an ideal setting for this focus because of the role dairy plays in its economy [36,37] and the state’s increasing reliance on migrant workers within its flourishing dairy industry. The risks that migrant dairy workers experience in these settings have been little explored. Effective occupational health studies among migrant workers are hard to do, due to the transitory nature of the farm workers, the difficulty in accessing these workers, their willingness to talk about occupational health issues (as many fear loss of job from such participation), and the lack of funding mechanisms to conduct oversight and good occupational health studies [12,38]. In this study we partner with the organization, Migrant Justice and utilize a triangulation of sources, including surveys and interviews, to provide a comprehensive overview of the occupational health experiences of migrant dairy workers in Vermont. Below we provide a brief overview of the Vermont dairy industry.

## 2. The Vermont Dairy Industry

Agriculture, dairy farming, and their associated idyllic landscapes, have long played a central role in the social identity of Vermont. While the role it plays in the state’s local economy is marginal at 1%, agriculture retains a strong footprint in the state’s land use with close to 20% of the state’s land invested in agriculture [39,40]. Dairy farms in Vermont contribute over 70% of agricultural sales and manage 80% of Vermont’s open lands, with much smaller contributions from agricultural crops to the local economy [40]. In 2019, the top five cash receipts were milk, miscellaneous crops, cattle, maple products and hay [41,42]. 

Nationally, Vermont is not a top dairy producer. The state produces less than 2% of all milk in the country, ranking 41st in net farm income among 50 states in the US [41]. Vermont, however, has the highest dependence upon a single commodity (milk) for agricultural revenue in the country, 63% of all milk produced in New England comes from Vermont, and the dairy industry brings in about $2.2 billion a year [41]. Dairy products make $1.3 billion in exports. However, the state has lost more than 90% of its dairy farms due to the continuous downturn in milk prices over the past 75 years, leading to the consolidation of small farms. A landscape history of Vermont dairy by Eric Kreig [43] notes that “dairy farms declined drastically from 11,019 in 1950 to 1459 in 2003, and to 677 in 2019,”as did the total number of dairy cows, which dropped from 257,000 in 1950 to 143,000 in 2005 and 125,583 in 2019 [43,44]. Despite these changes, the total amount of milk produced statewide increased significantly, due to higher yield per cow—2.5 billion pounds of milk in 2010 to 2.6 billion in 2019 [44]. Vermont dairy farms are largely medium to small entities with 185 Animal Feeding Operations (200–699 cows) and 25 Concentrated Animal Feeding Operations (over 700 cows) [45]. The growth in the industry with fewer larger farms producing more milk than ever has necessitated the adoption of capital-intensive production and intensive feeding technologies instead of grazing. High levels of year-round milk production are also sustained by growing greater amounts of corn for winter feeding [43].

While Vermont is known for its renewable and green energy infrastructure, its higher number of organic farms than in other states, about 80% of Vermont’s dairy farms are conventional [46]. Vermont’s reliance on confined, non-grazing dairy production means that dairy farm workers are exposed to diverse bioaerosols, antimicrobial residues, and chemicals. Nitrogen fertilizer use has increased by 17% and pesticide use by 39% between 2008 to 2012. 96% of corn grown to feed the cows are GMOs, which has not reduced the herbicide use, glyphosate, which has doubled since 2002, peaked in 2016 and has slightly gone down since then [47]. Vermont has the highest rate of glyphosate use in the New England region [48], Atrazine and metolachlor are the other top herbicides used in Vermont within agriculture [47]. Agriculture also represents 80% of total antibiotic use in the US. [49]. In 2017, the US mandated that antibiotics that are medically important for humans can no longer be used to promote growth or feed efficiency in food animals. This resulted in a 33% reduction in sales, but therapeutic use in food animals has more than doubled [50]. There is no state monitoring of antibiotic use on farms in Vermont, although antibiotic-use violations have been identified since 2010 [45], demonstrating a need for improved antimicrobial stewardship. Furthermore, Nitrous oxide and methane also increase greenhouse gases and exacerbate climate change, which may further necessitate the use of pesticides, fungicides and herbicides to control invasive weeds and pests [51], further increasing the risks of chemical exposure for farm workers in the future. Vermont’s dairy industry though smaller in size still poses a wide range of risks to its workers, this research examines the occupational and health risks to its most vulnerable population—the migrant dairy workers. 

## 3. Migrant Dairy Farm Workers in Vermont

Immigrant labor is an integral component of the Vermont dairy industry, especially as it expands and modernizes [36,37]. A 2010 survey reported that 75% of farmers believed there was a labor shortage and 37% thought that Latinx migrants could sufficiently fill that gap [52]. As of 2016, approximately 1000–1500 Latinx migrant dairy workers are in the state, 90% in this group are potentially undocumented [36,52], although accurate estimates are lacking due to the legal risks to employees.

Baker and Chappelle’s surveys [37] involving 120 Latino workers on 59 Vermont dairy farms between 2009 and 2011 showed that Latinx workers were young and predominantly male; 93% were Mexican and 7% from Guatemala. Of those surveyed, 89% only spoke Spanish and 4% reported being able to speak English well. Many of the workers were found to have little to no experience with the United States’ agriculture sector, and the laborers were highly transient, with 50.8% having been on their current farm for no more than 1 year and 8.3% for more than 3 years. Of these migrant dairy workers surveyed, 91.6% were milkers and worked a mean 64.5 h per week with a median hourly wage of $7.75 an h. All of the workers surveyed lived on the farm [37,53]. Undocumented migrants have become integral to keeping smaller farms open, and low wages and relaxed working conditions are considered to be the cost of producing milk at record levels and to sustain the industry in a highly competitive milk market.

Few studies have been conducted examining the health and safety of Vermont dairy farm workers. Baker & Chappelle found the most common health issues to be back/neck pain and mental health issues [37]. Isolation and fear of Immigration and Customs Enforcement (ICE) were also found to be primary barriers to healthcare. Wolcott-MacCausland found that barriers to healthcare access for Vermont workers in border counties led to self-medication and a dependency upon employers for care [54]. More information on the unique occupational and environmental risks to migrant farmworkers in Vermont is needed; the current study fulfills this gap. 

## 4. Methods

This research draws on a triangulation of sources including analysis of internal documents of the organization Migrant Justice, surveys and interviews they conducted with migrant dairy farmworkers as well as interviews with relevant community organizations that work closely with the dairy farm workers in the state to characterize the occupational and health risks experienced by migrant dairy workers in in this study. 

We realized that conducting a full-fledged occupational health study among migrant dairy workers without adequate resources was next to difficult. Hence, getting consent to access the internal documents of Migrant Justice was deemed the best approach in conducting the study. Migrant Justice is an organization founded and led by farmworkers who have worked extensively with migrant dairy workers across Vermont to “build the voice, capacity, and power of the farmworker community and to engage community partners to organize for economic justice and human rights” [55]. First, we got special permission from the board of the organization Migrant Justice to access and examine their internal documents—the surveys they have gathered every five years (2014 and 2019) and the in-depth interviews that they conducted with migrant dairy farmworkers (2019–2020), along with the other relevant documents they have produced on occupational and health risks to conduct the first part of the study. Migrant Justice started doing the surveys in 2014 with migrant dairy workers to better understand their priorities and safety concerns in order to best represent their needs in the society [56]. We analyzed the health and safety surveys (*n* = 107) conducted with migrant dairy workers between September 2018 and April 2019 and the 176 surveys conducted with dairy workers in the summer of 2014 (here on referred to as the ‘2019 and 2014 Health and Safety survey’) [57]. This study also analyzed the interviews with 14 migrant dairy farmworkers and a focus group interview with five individuals between 2018–2019 [58]. 

We reviewed the Migrant Justice study materials and found that it was professionally designed and it examined relevant occupational and health issues (especially the 2019 health and safety survey and the 2019–2020 interviews conducted with migrant dairy workers). These survey instruments though were not consistent between 2014 and 2019. The 2014 surveys focused more on basic worker rights, safety, and immigration issues, while the 2019 surveys extensively explored occupational health issues. Both the surveys were administered by trained farmworkers who carried out peer-to-peer surveys by meeting with workers on farms, at churches, etc. The peer-to-peer approach is an important methodological approach that has shown to increase participation, and to recruit difficult to reach population [59]. The peer-to-peer approach used in this case are likely more effective in recruiting migrant dairy workers who are otherwise unlikely to participate in surveys conducted by outsiders, as many migrant dairy workers live on the farm, do not speak English, are largely undocumented, are supervised throughout the day, and work between 10–14 h a day average. The 2019 surveys, (more relevant to this work as it focused more on occupational health issues) solicited information about living and working conditions on the farm, exposure to chemical/animal/machinery risks, training and safety precautions, health and mental health issues, and access to medical services [57]. The farmworkers surveyed came from dairy farms across the state. The migrant dairy worker interviews were conducted along with the interns at the Columbia Law School Human Rights Clinic, who could be considered a neutral third party. Notes were taken at these interviews. Not everyone consented to audio recording. The interviews similarly gathered information on health and safety conditions on dairy farms in Vermont [58].

In addition to reviewing the relevant Migrant Justice documents, we conducted key informant interviews (*n* = 10) with community organizations that work with migrant dairy workers across Vermont. Vermont being one of the least populated state in the US, there are not many organizations working on migrant dairy farmworker health and rights. All interviews were conducted in person between mid 2019–2020, except for one which was conducted by the authors via video-conferencing software due to the outbreak of the COVID-19 global pandemic in 2020. The key informant interviews were semi-structured, lasting an hour to an hour and a half. The interviews explored key environmental, social, and health problems among migrant farmworkers in Vermont. All participants interviewed in this study were over 18 years of age. All study protocols followed the Institutional Review Board protocols.

Triangulation of these three key sources of data (key informant interviews, surveys and interviews with migrant dairy workers) presented here together facilitate cross-verification of the sources for the consistency of findings and the study presents a comprehensive understanding of themes presented across these three databases [60]. The qualitative data were analyzed using NVivo 12, and the descriptive statistics of the survey data were analyzed using excel and integrated with the qualitative data. We worked across common themes to identify deviating perspectives, and to assess the credibility of the findings. The themes derived were influenced by the theoretical frameworks of precarious work and health inequity [61,62]. Precarious workers are defined by International Labor Rights Forum as “those who fulfill permanent job needs but are denied rights given to a permanent employee [62,63].” Instead, they are subject to insecure employment, systemic inequality, discrimination, disproportionate risks, and dangerous working conditions as it is in the case of many migrant dairy farm workers in the US. Many scholars define precariousness as a new form of regulation or domination stemming from neoliberal economic and market policies that favors downsizing and outsourcing, weakening of collective bargaining and labor regulations, and externalization of risks [64,65]. In particular, agricultural exceptionalism since the 1930s excludes farmworkers from most labor regulations, which perpetuates structural inequities. Farm workers are not included in regulatory laws such as the National Labor Relations Act and the Fair Labor Standards Act, making it possible for employers to violate labor standards for training and compensation [3].

While migrant dairy workers experience many of these precarious conditions at work, they are also essential workers as made apparent with the onset of COVID-19 [66,67,68]. While they are considered essential workers, the precariousness of these jobs introduces many more social vulnerabilities and risks. The essential nature of these occupations compounds these risks by exposing them to other public health hazards while having inadequate access to healthcare, insurance, transportation, and housing. Migrant dairy workers are thus precarious essential workers, whose day to day occupational risks need to be better characterized to better address the preventable health risks.

## 5. Results

Migrant dairy farm workers face a variety of work-related health risks and barriers to wellbeing. In this section, we characterized the following experiences of migrant farm workers: (1) working conditions on dairy farms, (2) social barriers to health and wellbeing, and (3) health outcomes self-reported by the migrant farmworkers.

## 6. Working Conditions on Dairy Farms

The working conditions on dairy farms characterize the work routines, wage inequality, harmful exposures at work, work and health and safety training. 

Work Routine: Both the survey and interview data showed that the work routine of migrant dairy workers varied among farms, and depended on factors such as the size of the farm, the number of workers, and the number of cows. The migrant workers interviewed completed a range of tasks, including taking care of the cows, carrying milking machines in manual farms, milking, connecting machines to the cows and the tubes that take the milk in mechanical farms, mixing cow feed, helping with the birthing of cows, taking care of the calves, putting hay in the den, giving foot baths, cleaning the premises and the gutter scraper, and fixing things on the farm on a daily basis. 

Key informants reported that migrant farm workers work long hours without enough breaks during the day. The 2019 survey results confirmed the key informant reports and identified that 94% of workers reported working at least 8 h, while 38% reported working 12 or more. Additionally, 24% reported that they did not have a break during their shift, and 30% reported that they work all seven days per week (see Table 1). This strenuous work schedule impedes the sleep schedule of about a quarter of the migrant dairy workers as well, with 23% reporting that they do not have time to sleep eight hours a night. While speaking about the long working hours, one community member said: “I mean it’s all over the place… there are guys who work 11:30 p.m. to 11:30 a.m., people who work 1 a.m. until 1 p.m., and some who work 7 a.m. to 7 p.m., and a lot of times they’re in situations where they can’t get a good night’s sleep. You put that together and that just seems like it ups the ante for danger.” The length of the work day depends on the specific needs of the farm. One community member reported: “I was talking to one guy who told me he’s supposed to work a 12-h day, but he helps out when the cows are giving birth, and so he was working 19 and 20-h days when a lot of them were having babies… he learned English so he could say to the boss you hired me for this many hours, but I’m working this many hours and not getting paid. They basically said, ‘Well go somewhere else’, and he did.” While this worker was able to find another job, not everyone is equally lucky, many also face termination if the workers fail to comply, or if they demand to be paid appropriately for their performed labor. 

Additionally, some farms are so small that there are only one or two workers, which increase the amount of work that they have to perform since there were no other workers to cover their shift. One community member gave a particularly harrowing anecdote: “There are workers who work eight hours, sleep for three, and then come work another shift. They never actually get to sleep for eight consecutive hours, for years.” This anecdote speaks to either the shortage of workers in the dairy industry or the inability of their employers to afford more field hands where the farmers themselves are struggling to make their ends meet in a market that continues to subsidize milk prices. The migrant dairy workers interviewed reported working a minimum of ten hours a day and a maximum of up to 16 h a day; most workers interviewed reported working 12–14 h in general. One of the migrant dairy farm worker said he milks up to 600 cows. Most migrant dairy workers interviewed said that they work without days off and do not get adequate sleep. Two workers out of the fourteen said that they get a day off a week, the rest did not. Two workers said that they got days off every fifteen days, at times in the afternoon to go grocery shopping. Some workers complained about the inability to engage in any social activities because of the busy work schedules.

Wage Inequality: In the 2014 survey, 40% of the migrant workers reported receiving less than Vermont minimum wage, 26% received no pay stubs, 20% had their first paycheck illegally withheld, 19% have worked for more than two years without a pay raise, and 40% received no days off. The 2019 surveys did not focus on wage inequality demonstrating that some aspects of wage inequality are being addressed by the organization. But community key informants note that wage theft is still a concern as many fear speaking up due to fear of losing job or being reported to immigration. The key informant emphasized that the problem was exacerbated along gendered lines: “That’s how we experience discrimination in the workplace, low wages. Especially if you’re a woman, you’re going to get the lowest possible amount, even if you’re doing the same or harder work than men, you’re going to get a really low wage.” The issue was raised by the migrant workers themselves in the interview noting that it was difficult to get a raise though the workload has increased. 

Harmful Exposures at Work: Both our survey and interview results showed that migrant dairy workers in the study were exposed to a wide range of chemicals. In the 2019 Health and Safety survey (Table 1), 96% of the respondents reported working with chemicals on the farm. The exposures reported were from chlorine (67%), footbaths (53%), iodine (51%), and acid (43%). In the migrant farm worker interviews, nine of the fourteen workers reported chemical exposures, four workers did not talk about it, and one worked in an organic farm. Exposure that workers were most concerned about resulted largely from iodine, acid, and formaldehyde. The activities that reported the greatest chemical exposure were cleaning and maintenance of the barn. Iodine is used for washing and cleaning, but its main usage is for cleaning the udders and teats of the cows. Community members reported that when using it in this context, it often splashed into workers’ eyes. One of the workers explained: “One time I was putting yodo [iodine] on the teats and the cow kicked me and I got yodo on my face. It got into my eye, and it really irritated my eye. I had a wrinkle or a crease on the surface of my eye in the corner. That stayed with me for about a month. It was hard to go outside because the brightness was painful. [Water] was able to clean it out, but the burning stayed a long time.” Chlorine is used mainly for washing and disinfecting the machines, and splashing occurs often onto workers’ faces, burning them. It was noted that chlorine is particularly dangerous if it gets mixed with other chemicals, such as ammonia. Similar to chlorine, acid is used for cleaning and is extremely dangerous, especially when mixed with other chemicals. One community member spoke about this and said: “With chlorine and acid, a lot of people don’t know how to use it or know the effects associated with it so they don’t wear protective gear such as a mask or gloves.” Footbaths for cow hooves were also another major source of chemical exposure for workers. Formaldehyde either in acidic liquid or blue powder form is used to kill fungi on the hooves, as hoof problems are frequent especially in big milking parlors. According to one community member, cows will walk through the footbaths in their loping shed and go immediately to the milking parlor, which spreads the formaldehyde around and gets it into the manure pit, which is then spread on the land, untreated. There is no filtering of hazardous waste. The dairy workers interviewed reported a range of impacts from formaldehyde from feeling dizzy, to vomiting, nausea, and nosebleeds: “We work with the powder that you use for the cow hooves. You put it into a tray or a tub and add water, but when you put it in, you have to hold your breath, because when you pour it, a fine powder comes up, and if you breathe it, then your nose starts to bleed.” Some workers reported using these chemicals daily.

Antibiotic use was another concern for dairy workers. One key informant who works closely with migrant workers reported that, on dairy farms, there is: “…routine antibiotic use. The second a cow goes dry, they go back to freshen up, routinely after lactation period. Almost all the big CAFO farms, the second lactations over, they give an antibiotic dose. Just for prophylactic purposes because these cows are in filth and standing in shit and urine water. So, it’s routinely used.” This community member also reported that although tolerances for antibiotic uses are set by the state, farmers can manipulate the tests to make sure that they do not go over the mandated tolerance. This was a concern particularly raised by the community experts more than workers themselves; the workers talked about using medicine on the cows but did not equate it as using antibiotics: “When a cow is sick and given medicine, you can’t add that milk to the rest or it will get contaminated. Workers mark which cows are sick, everyone does this in the process of their work, and you have to be careful to pay attention to that [or risk getting in trouble].” In the 2019 Migrant Justice survey, 26% reported harm from exposure to medicine or syringes.

Community members that were interviewed made it clear that working on a farm meant being in constant contact with chemicals, not just from milking, cleaning, and disinfecting, but also from pesticides applied on the dairy farms. Some community members expressed frustration with how the state responds to the excessive/dangerous use of chemicals on farms, especially when farms are found to be in violation of a law regarding chemical usage. One community member mentioned that he filed a complaint with the agricultural agency because he a saw a farmer: “Set up a make-shift place where he was mixing all his pesticides. And he had all these containers, it was out—it was that rainy period in June. It was all just soaked wet on these cardboard boxes of atrazine! glyphosate! metolachlor! All the containers just thrown, getting rained on.” He continued, that the agricultural agency took a month to respond to the claim, and by the time someone came out to look into it, it was gone. The cavalier practices further increases exposure to farm workers, putting their health and lives in danger. Several community members expressed concern that the water at the farms might also be contaminated due to the “heavy-duty fertilizers” and range of other chemicals that could seep into the water system, and mentioned that many of the workers do not drink the tap water on the farms, drinking bottled water instead to avoid contaminated water. One of the farmworkers interviewed confirmed that water in the farm had a bad smell and thus the worker drank bottled water. Several community members also expressed that the workers are largely ignored when the public thinks about agriculture in Vermont, as the state is perceived as a “pure place,” versus the reality of hazardous chemicals used in agriculture that workers on the farms deal with every day. 

Access to Safety Equipment: Access to safety and protective equipment is lacking on many dairy farms. In the 2019 Health and Safety survey, 66% of the respondents did not have protective masks, 71% did not use safety glasses or winter gloves, and 75% did not have aprons. About 50% reported that they would feel comfortable talking to their employer about health and safety. One community member gave an example of a worker who would work on his day off to clean the machinery using heavy disinfectants without gloves or a mask (because nothing was provided for him) in order to get a bonus. This type of behavior was encouraged, with no real regard for the health of the worker. Another community member spoke about the dangers of being exposed to chemicals, even with protective equipment, making the lack of it particularly concerning: “When dealing with toxic chemicals that we know are designed to kill life, protection can reduce exposure but one is still exposed to these chemicals in smaller doses daily in so many ways. It adds up.”

Access to safety equipment has been lacking for farm workers in general, but it has been particularly salient during the COVID-19 pandemic. When asked about how work is being impacted by this global emergency, one community member said, “Farm workers have never had any benefit or access to health care since the 1930s when farm workers were excluded from labor laws. There’s always been a shortage of masks and gloves, we already don’t have that on the farm. We have been doing this a long time. With COVID-19, things are going to continue. We have to milk cows, our schedule will continue, there is no health care for us, we cannot take a day off.” The current global health emergency reveals both how lacking protections for farm workers are and how essential they are to keep workers safe and to keep the food system running.

Work Safety and Health Training: Many migrant farm workers are unaware of the risks posed by the chemicals they use. The 2019 health and safety survey showed that 67% of migrant dairy workers did not know the risks associated with chemical use, and 51% did not use chemicals in accordance with instructions. Training on farms has the potential to curb the hazards of the environment, but access to adequate training is lacking. Only a quarter of respondents reported receiving training on chemical safety on the farm, half reported receiving training on animals safety; and close to 45% reported receiving training to safely operate the machinery and other equipment on the farm. Training on biological risks (7%), environmental risks (12%) and sexual harassment (5%) were far lower. 

Additionally, it is important to consider that much of the training received was in English, unless a co-worker translated or trained them. And if co-workers are training other workers, they are not being compensated for their time and expertise. Ten of the 14 migrant dairy workers interviewed said that they did not receive work training and were largely trained by co-workers or learned by copying others on the job to use milking machines, feeding and giving medicines to calves. Even the worker who worked in the organic farm said that she received no direct training and learned only from co-workers. But the worker also stated that their employer was very receptive to worker needs and has provided boots, gloves, aprons for safety. Workers have suggested that videos are helpful in training, but there are not many available. Overall, a community member added: “A lot of the time when the training is done by the boss, even if it’s with the best intentions and hand signs, you don’t really get to ask questions, and that is really important. Without questions, you can’t ask about the risks that lead to a lot of accidents on the farm.” While one can learn to conduct work through practice, lack of adequate training may increase the chance of exposure to hazards and risks at work. 

## 7. Social Barriers to Health

In addition to the physical and occupational hazards that workers face, they are also subjected to multiple social hazards including immigration pressure, language barriers, access to adequate housing, access to healthcare, and access to transportation, which are detailed below.

Immigration Pressure: The 2019 Health and Safety survey reported that 37% of workers were concerned that their boss would call immigration services (Table 1). The 2014 health and safety surveys noted that 32% of the workers felt that they were not treated equally as US-born workers. Results demonstrate that immigration remains a great concern among migrant dairy farm workers, many of whom are likely undocumented. Community members reported that workers live in constant fear of racial profiling, especially because many of the farms are within 25 miles of the northern border in the Northeast Kingdom and Franklin County, a particular additional threat due to the larger presence of US Customs and Border Protection (border patrol) in the state. The key informants noted that the workers were often afraid to leave the farms because of this threat. This perceived risk is leading to extreme isolation, generating additional power dynamics between farmer and worker—with workers fearing speaking up about work hours or wages because the farm can call border patrol. This observation was confirmed by a migrant dairy worker report: “We cannot leave because of immigration, we are near the border. When we need something personal we cannot get it. We have to pay someone to go get it for us. … not being able to leave the farm makes me feel stressed, feels locked up, caged and stressed. A year ago, could leave, but now have stopped leaving after a few coworkers were arrested. …it is tough but we know we came here to work and go back home. Yes, if someone would take us would have more trust/would feel more protected.” 

This inability to leave due to immigration issues is particularly dangerous during health emergencies. One community member spoke about cases if an emergency occurs, many workers will choose to stay home, rather than potentially endangering themselves by going to a hospital. The member noted a case involving immigration being called at the hospital—the workers could not speak English and hospital staff called border patrol. He said: “People stay home and hope that they heal, you know, because you’d rather stay home and support yourself than go and expose yourself and go to prison because no one’s going to take care of you in prison, and then your family is the one suffering.” The community key informants also said that in order to avoid border patrol, some people will travel three or four hours farther south just to access health services. He gave an example of a pregnant woman who had to take precautions: “…so she was going to the hospital for her checkups and she started noticing that the border patrol started showing up to her checkups, so she stopped going and had to arrange for someone to come to her house because of fear that she would be detained while pregnant and imprisoned.” Fears of being detained were not unfounded, especially during the Trump administration. One community member said that, as people were speaking up against the administration and farm worker conditions, leaders were being targeted, especially members of Migrant Justice. Two people were arrested during a Milk with Dignity campaign, when people marched 13 miles to the Ben & Jerry’s headquarters.

Language barriers: Language poses significant problems for migrant workers. In a survey conducted among migrant dairy workers in Vermont between 2009–2011 [37], 89% of the workers spoke Spanish and just 4% reported being able to speak English well. One community member reported incidents of harassment of workers who did not speak English. Another member mentioned that during a forum held for migrant dairy farm workers, many workers spoke about the urgent need to learn English. Many workers attempt to learn from fellow laborers, or learn to communicate through other cues and hand signals, or sometimes limit communication. Both lack of adequate training and lack of English proficiency increases the health and safety risks to migrant workers. These concerns were also highlighted in the migrant dairy farm worker reports: “Yes, he [employer] does not understand Spanish, or understands very little, and he expects us to understand English, and when we don’t, he gets mad.” This worker also talked about translation services available in some farms: “There was someone who came in and help us understand him, and he does a good job with that.” However, it would be hard to have a translator around all times when needed. Additionally, lack of English proficiency also impacts access to transportation. One community member reports that the process of getting a driver’s license in Vermont is extremely difficult unless you have a good understanding of English. Language barriers also pose specific challenges to accessing relevant information and specific state-based recommendations made on the pandemic as well.

Access to Adequate Housing: Poor housing is a significant issue for migrant dairy farm workers in Vermont. Community members identified that there is no real standard for farm worker housing, and while conditions are not always bad, these vary wildly, but most of them fall in the lower end: “There’s a group of people I work with that live with the cows in the barn. One of the people has no windows… We cut a hole in the side of the barn and put the exhaust out, so at least he could sleep,” The 2019 Health and Safety survey results show that these statements are no exaggeration: 20% reported living in or near the barn and 7% reported lack of heating in their housing (Table 1). In the 2014 Health & Safety survey, 30% reported living in overcrowded homes, 16% slept on a living room sofa due to overcrowding, 35% of the workers noted that the housing needed major repairs, and 15% had insufficient heating in the house.

Sleeping in the barns is not uncommon. A migrant dairy worker reported living in an area where the tractors were stored. Additionally, the barns get extremely hot in the summer and cold in the extreme weather of the winter. In one case, a worker had to wrap their whole apartment in plastic because “the wind was just ripping through it.” A migrant worker living above a barn reported: “We don’t have a heater. It is hard to be in the living room or the kitchen because you tremble from being so cold. Housing is the most important thing I would like to see changed. It is not a good house to live in. The kitchen doesn’t really work and there is nothing there. It is nasty in the summer, because all the smells from the cows go up into the rooms above and there is no ventilation. Even with air fresheners you can’t get rid of the smell. …We need a better place to rest, and there is no dignity or privacy in the house. It is not comfortable to have someone over and for them to shiver in the kitchen. So we don’t invite anyone over.” Another worker who lives on the farm reported: “The smell of the farm is pervasive throughout the living quarters, and the workers are exposed to the constant noise of machinery. This prevents being able to sleep continuously at night.” The worker also reported that they had to buy their own heating unit as there was no heating in the house. 

The barns and some housing provided tend to be dirty and infested with bugs and rodents. One community member mentioned a roach infestation in the housing of one of the farms that had been unresolved for years. One of the migrant worker reported that rats are a problem because they also eat the food: “we have rats and cockroaches. And the floor of the house is just bare concrete. …we don’t have pantry for food that doesn’t go in the fridge. We put it on open shelving, and rats and mice get in there. …everything that doesn’t go in the fridge, like bread, rice, beans, you need to hide it so that they can’t get to it. You put it in the bedroom or hang it from the ceiling. The wooden wall in the living room is also broken, and they live and breed in there. I’ve killed about twenty of them, but they keep coming back.” Another migrant worker reported that his housing is old and had bedbugs which impacted his ability to sleep: “The farmer bought some powder to use which has not been useful.”

In addition to poor housing conditions, some workers are faced with hidden housing costs that they did not know they would have to pay. One community member spoke about how since over 90% of workers are undocumented, housing needs to be provided, which is what attracts a lot of people to work on farms in the first place. However, some workers have noticed that farmers take out reductions on pay stubs. 

Access to Transportation: The lack of transportation isolates many migrant workers from society and they have to rely on others to travel outside of the farm to a convenient store, or health center, or community centers. Community members noted that the lack of transportation or ability to get a driver’s license limits access to health services and to get assimilated into the community. In Vermont, undocumented immigrants can get a driver’s license since 2013, but getting to the appointment is difficult for workers given their complex work schedules and lack of rides. Public transportation is poor in rural areas and most migrant workers do not own a vehicle. One community member who spent time in a food pantry, asking migrant workers what their barriers to health were, reported that transportation was a bigger concern to them than food, even though they were in a food pantry. Some workers also felt more comfortable going with others in the community because of racial profiling. While some transportation services are available to migrant workers through concerned advocacy organizations, they may still lack guaranteed transportation during an emergency, or when it is most needed.

Access to Health Care: In the 2019 Health and Safety survey, 63% reported having access to doctor/medical services, 34% reported having access to first aid, and only 16% reported having health insurance (Table 2). Farm workers have been excluded from health services by law and do not qualify for free health care or Medicaid, despite the dangers in the job. Because of this, when workers get sick or injured, they do not seek help, in some severe cases their employers pay for the health expenses but not all health expenses are covered. Community members reported that the emergency room is extremely expensive for workers, and so largely, workers stay at home and hope that they heal on their own. In the migrant farm worker reports, two of the fourteen workers reported that the employers paid for injuries that were serious, three reported no help from the employer to cover medical expenses, and two reported that the employers do not take their health concerns seriously. Despite these issues, community members have noted some improvements. One person spoke about open door clinics that are helpful because they have Spanish services and some community services provide access to transportation. Vermont is also one of the few states to offer stimulus funding for undocumented immigrants [69]. 

## 8. Health Outcomes

In this section, we report the accidents and injuries, musculoskeletal risks, health risks from chemical exposures, and other chronic conditions experienced by the migrant dairy workers.

Accident and Injuries: Many of the injuries reported in the 2019 Health and Safety surveys were from being hurt by an animals (78%). 44% of the respondents reported harm from unsafe animal gates, and 70% reported being hit or kicked by an animal, 8% reported being bit by animals (Table 2). The 2014 dairy worker survey showed that 30% of the workers suffered from a workplace injury or work-related illness. A worker reported that he needed to get three months rest due to a back injury and fracture by being stampeded by a cow. Another worker talked about being hit by a cow in the chest and he could not breathe without it hurting because the chest was so inflamed. Accidents were also reported from use of machinery. In the 2019 surveys (Table 2), 22% of workers had been harmed by milking machines, and 44% by unsafe gates or doors not closing or being crushed. In the migrant worker reports, a worker explained: “I get kicked around twice a month by cows, this was due to lack of training. And the manual gate makes it really easy for workers to get kicked by cows often. … we have to push the cows one by one after they’re done milking and open the gates with our hands [than electronically]. And when you push them off that’s when there’s a lot of risk of getting kicked or injured.” Seven others interviewed reported similar issues. Another worker was hit by a cow while opening gates which sliced open his lips and the worker could not eat or talk properly for a month, and he needed to have braces put in his mouth. The workers also talked about being hit by milking machines when the tubes are dropped down and pulled back up mechanically, injuries from carrying heavy machinery, and falls. Some were concerned about the lack of maintenance of the machines that were dangerous to work with: “There is a platform that raises and lowers cows in parlor. It is run on hydraulics. I told him that there was a leak in the hose, that could cause a failure. One time it fell suddenly and it crushed a cow.” Both the community experts and migrant dairy farm workers said that accidents frequently occurred from slipping, because the floors of the parlor are always wet with milk. In the winter, the floor freezes and makes it more slippery in the parlor. There are also a lot of trip hazards. The wet floors coupled with trip hazards make carrying heavy machinery particularly dangerous. 

Musculoskeletal Risks: Musculoskeletal risks result from carrying heavy machinery and doing repeated motion for an extended period. In the 2019 Health and Safety surveys, 77% of workers reported being harmed from a musculoskeletal risk (Table 2). Of these workers, 82% also reported having pain in the back or neck from moving or carrying heavy things. Additionally, 58% of workers reported being harmed by repetitive movement and 73% also had pain in the back or neck from repetitive movements. A community member described: “You’re in that milking position for years basically, every single day… Lots of harm from musculoskeletal things.” One of the workers who survived being stampeded by a cow said that he continues to have problems from it especially when it gets cold: “Have pervasive pain in the bones from the cold.” Another worker reported pain in the abdomen from heavy lifting: “Cause of abdominal pain was not found and it continues to hurt from time to time. Some days I am so tired and hurting a lot that I have to take painkillers to keep working:” A female migrant worker shared that she has hip injury from carrying heavy buckets of milk. Often, these musculoskeletal injuries are not taken seriously or are disregarded by the workers themselves since they have to continue working to earn a living, but these are also ignored by the employer. A worker noted that his employer is disrespectful and mocks workers and does not take worker injuries and health complaints seriously. 

Health outcomes from chemical and biological risks: In the 2019 Health and Safety survey, around 83% of workers reported experiencing harm from a chemical or biological risk. Risks reported were from manure (44%), footbaths (55%), iodine (51%), feed and sawdust (49%), acid (43%), medicine and syringes (16%), and insecticides (16%) (Table 2). In the migrant dairy worker interviews, half the workers complained about health concerns due to exposure from iodine, acid, or formaldehyde, with effects ranging from irritation to the eye from accidental splashing, nosebleeds, breathing difficulty, nausea, and allergies from chemicals, saw dust, or the cow feed. The 2019 survey results (Table 2) noted itchy eyes (49%), coughing (47%), headaches (50%), skin rashes (36%), skin burns (32%), allergies (26%), nosebleeds (32%) difficulty breathing (27%), and vision problems (22%). These reports highlighted widespread acute and chronic health exposures and more immediate health outcomes. 

Psychological Health: About 67% of workers reported one or more concerns related to mental health in the Health and Safety survey (Table 2). The most prevalent reports were feeling stressed (64%) and feeling depressed (40%); and 29% reported stress about losing their job. One community member mentioned that that some migrant workers felt isolated and desired to socialize with the broader community. Sexual harassment is another issue on farms that leads to poor psychological health outcomes. One community member reported harassment occurring from farm management, impacting mainly women and the LGBTQ+ community.

## 9. Discussion

Occupational health issues of migrant farmworkers have been characterized since the advent of the environmental justice movement and are among the central environmental justice issues of interest to the Hispanic community [70]. This study characterizes the widespread inequities in the dairy industry in Vermont that disproportionately impacts migrant workers.

Vermont’s identity is heavily influenced by the dairy industry, as an idyllic state with rolling hills, rural charm, Cabot Cheese and Ben and Jerry’s ice cream. Even the corporate entities, such as Unilever-owned Ben & Jerry’s Homemade Holdings Inc., are widely portrayed as symbols of environmental responsibility, sourcing from ‘happy cows.’ This was however challenged in 2018 by the Organic Consumers Association (OCA), which sued Ben & Jerry’s for deceptive labeling, marketing, and sale of its ice cream, since they source milk from large, concentrated animal feeding operations, where cows are raised in confinement their entire lives, relying heavily on genetically modified feed crops, pesticides, and routine use of antibiotics [71]. Some activists who opposed the diary corporation were subpoenaed by the law firm Shook Hardy, who was also the legal aid for Philip Morris and Monsanto, as a legal scare tactic in relation to the OCA lawsuit [72]. OCA won the case in 2020, and Ben & Jerry’s can no longer claim that their ice cream comes from ‘happy cows’ [71]. Similarly, the state that is dominated by the dairy industry has also not been forthcoming about the true impacts of dairy farming—its widespread reliance on chemicals, and antibiotics in Vermont. As one of the community partners have said, “You cannot touch the dairy industry in Vermont.” The industrial and state practices of invisibilizing risks also enforces racial capitalism within the dairy industry that disempower and erase the experiences of Hispanic workers in Vermont. Vermont is 95% White; issues related to racism or racial capitalism are not easily visible or addressed unless these are alarmingly visible issues. The plight of migrant dairy workers similarly were not visible until the death of the dairy worker José Obeth Santiz Cruz, who was strangled to death by a mechanized gutter scarper in 2009 [73]. This tragic event led to the formation of a solidarity collective and the formation of Migrant Justice that now fights for economic justice and human rights of migrant dairy farm workers in Vermont.

Since 2010, comparison of the survey results from 2014 and 2019 Health and Safety Surveys (Table 3) shows a slight improvement in work practices such as breaks during the shift and time off during the week or having better heating in the house. Workplace injuries of 30% reported in 2014 in this study is consistent with injuries (29%) reported by Menger et al. in Colorado in 2019 [74,75]. Menger et al. also reported that 64% of the injuries were animal related, this study however reported higher rates of harm from animal related risks, this increase may be due to the difference in wording between these instruments *injury* vs. *risks*. The 2019 surveys in this study detailed biological and chemical risks of 83% which compared to 53% of biological risks and 47% of environmental risks in Menger et al. [74,75]. Over half of the respondents in this study were exposed to chemical risks such as iodine exposure and formaldehyde, and close to half of them had acute exposure reactions such as itchy eyes, cough, and headaches. These outcomes are hard to ignore when reported by half of those surveyed and is consistent with other studies on the acute health outcomes among migrant dairy workers in Vermont and elsewhere [1,30,35,37]. The information on chronic issues is lacking because of the transient nature of the occupation, and the long-term impacts are especially ignored, contested, and unaddressed, and are the specific casualty or externality of neoliberal work ethics [12,61]. Risk can also be viewed as a stressor, which can exacerbate poor health by weakening the body’s defense against these challenges making people susceptible to other threats, including COVID-19 which also attack the respiratory system. 

The relative risks in these cases are amplified due to lack of care—the risk of exposure to harmful chemicals are underplayed or not taken seriously enough by the industry. Of the survey respondents, 75% said they did not receive training on chemical safety, 92% did not receive training on biological risks, and 88% did not receive training on environmental risks. In Colorado, Menger et al. reported that 40% received health training related to zoonotic and infectious diseases, which shows that other states with bigger dairy industries may be doing a better job of providing adequate training [74,75]. These results demonstrate that there is little conversation on “exposures to risks” from bioaerosols, pesticides, formaldehyde, and antibiotic use in the state. By “invisibilizing risks,” workers are perceived as expendable and risks as inherent to participating in these jobs, though these risks have long-lasting health implications.

The high occupational health risks also stem from racial discrimination. In Vermont, the geographic isolation of farms, high rate of undocumented workers in dairy farms, and the threat of immigration for the Hispanic migrant workers restrict their mobility, increases their exploitability and the precariousness of migrant dairy farm workers as observed in this study as well as others [36,37,52,53,54]. The threat of deportation have shown to contribute to poor mental health as well [37,54]. Low income and race in the US continues to be the most significant predictor of poor health outcomes among vulnerable groups and Latino population suffer one of the highest health disparities in the US [76,77]. The Hispanic population are also one of the most affected by COVID-19 [78]. This may be related to the fact that many Hispanic people work in essential jobs that cannot be practiced from home but are also in precarious jobs, with higher harmful exposures, health risks, inadequate training and work benefits which increases the vulnerability many fold. 

Understanding how various elements within migrant dairy work and health are interconnected is essential to more adeptly address these issues through community and policy interventions. Many of the risks and health conditions that the workers face can be eliminated with good labor practice—by providing fair wages, adequate health and safety training, access to health services, and transportation to workers to seek care when needed instead of transferring the employer responsibilities to workers to maximize costs. Studies have shown that health literacy is crucial to protecting health outcomes [35]. Current literature shows that training, fair wages, and health care access are important resources for avoiding poor health outcomes [28,29,30,31,32,55,79]. In Vermont, the work of Migrant Justice has led to the improvement of working conditions in dairy farms throughout the state. They brought forth legislation in 2013 which provides driver’s license to Vermonters regardless of immigrant status. They have been organizing to build public pressure to prohibit racial profiling, and to free members detained by Immigration and Customs Enforcement and by Customs and Border Protection [55]. While these projects are underway, the 2019 survey results show that more needs to be done to address occupational risks in dairy farms and to improve the health and safety of workers. The health and safety training needs to be more rigorous and provided in languages understandable to workers. Universal access to healthcare for essential workers would improve access to healthcare and mitigate the immediate health concerns these workers face. While training and health access might improve the health outcome of the workers, the industry still needs to address the use of harmful chemicals and antibiotics in agriculture that threaten the workers every day. The migrant farmworkers put their bodies on the line each day to provide essential services. 

The results in the study are not generalizable due to the multiple limitations within the methodologies employed in the study. Our study combined approaches of interviews and surveys, and analyses of secondary data from Migrant Justice, to be less invasive in our data collection practices. Studies on occupational health among migrant farm workers are especially hard to conduct. For this reason, relying on data gathered by community organizations engaged in advocacy of the farm workers for years, and which are trusted in the community, was considered the best option. However, the health and safety surveys conducted by the organization are not longitudinal studies designed and implemented by experts in the field to test specific occupational health outcomes, but have been initiated to better understand the risks and needs of the people to advocate for a better future for migrant farm workers. Hence, the risks and the health outcomes experienced by the manual laborers in the farms were not consistently documented, nor fully characterized. Consistent and standardized monitoring and documentation is essential to fully understand the range of risks migrant workers face in the dairy industry in the US. 

## 10. Conclusions

This study characterized the occupational and health experiences of migrant dairy farm workers, who are essential workers. This population is integral to the food system, image, and economy of Vermont, and yet they face considerable inequities stemming from long hours at work, exposure to chemicals, bioaerosols, and antibiotics. They receive inadequate health and safety training, are not provided adequate health and safety equipment, resulting in accidents, injuries from animals and machinery, and suffer other health outcomes ranging from respiratory issues, headaches, vision problems, musculoskeletal pain, depression and stress. As the state addresses the labor shortage in Vermont dairy by hiring more immigrant workers, more attention should be paid to the existing working and living conditions of vulnerable migrant dairy workers, so a diverse, equitable, and just sustainable dairy industry can be built in Vermont. Better health and safety training and universal healthcare access to essential workers are required to protect basic human rights. Additionally, reducing harmful practices and exposures in dairy farms would benefit the workers as well as the environment tremendously. Overall, the pandemic has made evident the critical work that immigrant laborers provide. The pandemic is a wakeup call to start doing things differently, to address the inequities within the workspaces of these essential jobs, and to stand in solidarity with the essential workers to protect their basic rights.

## Figures and Tables

**Table 1:** 2019 Health and Safety Survey: Working conditions in Vermont dairy farms.

Work Schedules	*n* = 81–107	%
Working for over 8 h	76	94%
Working for over 12 h	39	38%
No time for 8 h of sleep	23	23%
No breaks of at least 15 min during shift	22	24%
No day off per week	31	30%
Ran out of food	24	23%
**Chemical Risks**		
Worked with chemicals	102	96%
Exposed to acid	47	43%
Iodine exposure from cleaning udders	56	51%
Liquid or powder foot baths/formaldehyde exposure	53	53%
Chlorine/bleach	68	67%
Do not know the risks related to the use of these chemicals	68	67%
Do not know to protect themselves when using these chemicals	57	57%
Use chemicals in accordance with instructions	35	34%
Do not follow instructions when using chemicals	53	51%
**Safety Hazards**		
Use protective masks	47	44%
Use safety glasses	31	29%
Had winter gloves	31	29%
Had aprons	17	25%
Adequate ventilation	94	88%
Felt comfortable talking to the employer about health and safety	54	51%
**Health and Safety Training & Services**		
Received training on chemical safety	27	25%
Received training on biological safety	8	7%
Received training on animal risks	53	50%
Received training on machinery risks	48	45%
Received training on environmental risks	13	12%
Received training on sexual harassment	5	5%
Concerned that the boss will call immigration	38	37%
**Housing**		
Workers living in or near the barn	20	20%
Lack of heating in the housing	7	7%
**Environmental Risks**		
Extreme heat	48	45%
Extreme cold in the barn	58	54%
Trip Hazards	51	48%
Slippery floors	54	51%
Hypothermia	14	24%

**Table 2:** 2019 Health and Safety Survey: Health risks in Vermont dairy farms.

Access to Health Services	*n* = 107	%
Access to a doctor/medical services	64	63%
Had first aid	36	34%
Had insurance	17	16%
**Accidents and Injuries**		
Hurt by animal-related risks	83	78%
Hit or kicked by an animal	75	70%
Bit by an animal	10	9%
Have contracted diseases transmitted by the cows	14	13%
Injuries from milking machines	24	22%
Harmed by unsafe animal gates	47	44%
**Musculoskeletal Risks**		
Harmed by machinery or musculoskeletal risk	82	77%
Harm from carrying or moving heavy things	34	82%
Harmed by repetitive movement	62	58%
Pain in the back or neck from repetitive movement	45	73%
**Harm from Chemical and Biological Risks**		
Experienced harm from chemical or biological risks	89	83%
Harm from manure	47	44%
Harmed from footbaths	59	55%
Harm from insecticides	17	16%
Harm from organic powder (food and sawdust)	52	49%
Harm from acid	46	43%
Harm from iodine	55	51%
Harm from exposure to medicine or syringes	28	26%
**Health Outcomes from Chemical Exposures**		
Itchy eyes	52	49%
Cough	50	47%
Headaches	53	50%
Skin rashes	38	36%
Skin burns	34	32%
Allergies	28	26%
Nose bleeds	34	32%
Breathing difficulty	29	27%
Vision problems	24	22%
Stomach problems	31	29%
**Mental Health**		
One or more concerns related to mental health	70	67%
Stress	58	64%
Depressed	41	40%
Concerned about losing job	30	29%

**Table 3:** Comparable results from the Health and Safety Surveys 2014 and 2019.

Comparable Results from Both Years	2019	2014
No breaks during shift	24%	29%
No day off per week	30%	40%
No time for 8 h of sleep	23%	23%
Lack of heating in the housing	7%	15%
Harmed by workplace injury or illness	-	30%
Harmed by musculoskeletal risk	77%	-
Experienced harm from chemical or biological risks	83%	-
Hurt by animal-related risks	78%	-

## Data Availability

The quantitative data and interview data among migrant dairy workers used in the study belongs to Migrant Justice. De-identified qualitative key informant data is available upon request.
